# Development of an On-Board Measurement System for Railway Vehicle Wheel Flange Wear

**DOI:** 10.3390/s20010303

**Published:** 2020-01-06

**Authors:** Pacifique Turabimana, Celestin Nkundineza

**Affiliations:** African Railway Center of Excellence, Addis Ababa Institute of Technology, Addis Ababa University, Addis Ababa 1000, Ethiopia; turabimanapacifique@gmail.com

**Keywords:** wheel flange, wheel rail contact, inductive displacement sensor, measurement, curvatures, wear

## Abstract

The maintenance of railway systems is critical for their safe operation. However some landscape geographical features force the track line to have sharp curves with small radii. Sharp curves are known to be the main source of wheel flange wear. The reduction of wheel flange thickness to an extreme level increases the probability of train accidents. To avoid the unsafe operation of a rail vehicle, it is important to stay continuously up to date on the status of the wheel flange thickness dimensions by using precise and accurate measurement tools. The wheel wear measurement tools that are based on laser and vision technology are quite expensive to implement in railway lines of developing countries. Alternatively significant measurement errors can result from using imprecise measurement tools such as the hand tools, which are currently utilized by the railway companies such as Addis Ababa Light Rail Transit Service (AALRTS). Thus, the objective of this research is to propose and test a new measurement tool that uses an inductive displacement sensor. The proposed system works in both static and dynamic state of the railway vehicle and it is able to save the historical records of the wheel flange thickness for further analysis. The measurement system is fixed on the bogie frame. The fixture was designed using dimensions of the bogie and wheelset structure of the trains currently used by AALRTS. Laboratory experiments and computer simulations for of the electronic system were carried out to assess the feasibility of the data acquisition and analysis method. The noises and unwanted signals due to the dynamics of the system are filtered out from the sensor readings. The results show that the implementation of the proposed measurement system can accurately measure the wheel flange wear. Also, the faulty track section can be identified using the system recorded data and the operational control center data.

## 1. Introduction

The wheelset of the rolling stock is an important component of a rail vehicle. It plays a role to keep the vehicle stability when it is in motion. A vehicle moving on a sharply curved rail track experiences the issue of having the wheel and the rail getting into two contact patches, between the wheel tread and the rail, and between the wheel flange and the rail gauge corner. This situation results in material loss from the contacting parts, which in return leads to the wear of the wheel tread and the wheel flange [1]. 

The wheel profile wear reduces the safety operation margin of railway vehicles. There is a possible lateral displacement of the load center on the track, higher lateral forces on the track structure, vehicle instability, passenger discomfort and risk of derailment [2]. A high level of wear of the wheel flange thickness is a danger to the railway vehicle when moving on a curve or on a straight track [3,4]. Therefore, the measurement and inspection of external and internal wheel profile parts, especially the wheel flange thickness, should be done with high accuracy to ensure the safe train operation [5]. 

When a rail vehicle is negotiating a sharp curve there is a large increase in wheel flange wear due to the high lateral forces at the contact patch [6]. Wear evolution on wheel treads and flanges has been classified into three main regimes: mild regime, severe regime and catastrophic regime [7]. Wear evolution steps are also identified as linear, nonlinear and undefined [8]. In a mild regime or linear regime, the quantified wear in terms of dissipative energy is proportional to the creep force. Severe wear is characterized by a nonlinear relationship between the creep force and increased contact forces because the creepage has become large and the creepage can no longer be approximated as a constant. In catastrophic regime the wear has an undefined relationship with creep forces. Therefore the wear in sharp curve will fall at least into nonlinear regime because of the increased creepages and contact forces between the wheel flange and the rail gauge [9]. 

Wheel flange wear inspections can be done visually, or by manual operations using hand tools like Vernier calipers, or using automated electronic tools. The use of hand tools is very simple. However, human errors are inevitably introduced because the measurements depend on the operators’ skills [10]. The existing modern measurement tools based on lasers and vision technology that are quite accurate can be very expensive. Also, most of the existing wheel wear monitoring systems are based on track side measurement systems such as “mechanical profile detectors”, “wheel profile detectors”, and “cracked wheel detectors” [11]. Monitoring the wheel flange status using these systems in real time requires the installation of many of these pieces of equipment along the railway which results in increased costs. Therefore there is a need to determining the wheel flange wear in real time with high precision and accuracy at less cost.

### 1.1. Wheel Wear Prediction Models

There exists an immense body of literature on model development for wheel tread and flange wear prediction, such as can be found in [12,13,14,15,16]. In these models, the track geometry and initial profile of the wheel and rail are used to determine contact points. The rail vehicle dynamics principles are used to calculate wheel rail interaction force parameters including creep forces, creepages, displacements, and the contact patch. The wear is calculated using energy loss at the contact patch or using an Archad wear model. Recent models included the flexibility of the track in wheel wear prediction models. It is found that the wear calculated using track flexibility is much less than the wear calculated using a rigid track [17,18]. Generally, there are a lot of parameters that play a role in the wear of a rail wheel. For example if the track flexibility has a role in the wheel wear process, the track longitudinal stiffness variations in a curved track will also be a factor via possible increased impact loads [19] and increased maximum rail deflections [20]. In addition, tract stiffness variations lead to varied track settlements [21], and hence, irregularities. Other factors such as surface roughness, and environmental conditions that could lead to track irregularities must also be accounted in the prediction models. In some cases railway researchers use stochastic analysis to predict the effects of such varied input factors on railway wheel and rail wear [22]. Although wear prediction models are validated by on site wear measurement [15,17], the aforementioned wear factors will alter overtime and therefore altering the wheel flange wear evolution process. In these instances, the earlier established prediction tools become inaccurate. In these instances, a real-time measurement system of wheel flange wear can be used to update and calibrate these prediction tools.

### 1.2. Wheel Flange Wear Measurement Techniques

The measurement technologies of wheel flange thickness wear has been studied by several researchers. Wheel flange measurement methods can be classified into: (1) manual measurement using hand tools; (2) measurement using electronic wheel profile meter when the railcar is at a depot (3) measurement using trackside electronic devices such as camera, lasers, ultrasonic devices and image processing devices, and (4) onboard measurement devices that use lasers and ultrasonic devices. Many of the related works can be found in Alemi et al. [23] and in Bernal et al. [24]. 

The fundamental measurement technique of the wheel flange thickness wear is by manual operation, using hand tools like calipers and steel rules [25]. This method has different disadvantages: need of calibration, dependence on the skills of the operator, low accuracy and precision, and the need to stop the vehicle operations for measurement purpose in depots or workshop. The wheel flange wear measurement methods have been improved [26] as a result of advancement of technology in railway systems all over the world. A portable electronic gauge [27] can measure the wheel rim diameter, wheel flange thickness and height, when the train is in the workshop, using simple operation. As to its advantages, it is rechargeable; the results do not depend on operator’s skills; and, it can record data in an electronic memory. However, the installation of equipment that support the operation delays the measurement process and it can be used only when the train at a standstill. On the other hand, different trackside measurement systems which can take measurements as the train passes around them have been developed. Among them, there is a digital image processing based trackside system that takes image of wheel tread and flange as the wheel passes across its lightening devices [10]. There is also an online detection system based on laser displacement sensor (LDS) [28] installed on the trackside. The wheel profile (wheel diameter, wheel flange width and height) was able to be measured. The system uses six 2-D LDSs and two 1-D LDSs. Due to the fact that the track has a right and left side, to prevent the frustration of the images, the system is divided into two groups; each one at each side of the track. These LDSs (1-D and 2-D) use the triangulation measurement principle. The system demonstrates rapidity, high accuracy and precision of measurement. However, it has to be installed below the track at each side; it exhibits costly components and maintenance; it has a complex construction. Another track side measurement tool using multi-camera and structured light vision system was developed by Zhan et al. [29] to measure the wheel profile of a rail vehicle passing in a tunnel. The system was installed on the track with a certain clearance. It is a complicated system with its 3-D caption and the measurement operator is the only who read the historical records of the wheel. There was an already developed track side system to measure the wheel diameter and tread profile [29]. It could take only the inner and outer profile measurements with the two pairs of structured lasers based on the cycloid constraint referred to the contact points at the two different positions. Its construction was not complex and had high stability and precision. The online non-contact measurement of the wheelset wear was also developed and installed on a table fixed near the running rail to avoid some disturbances from vibrations [30]. The images of the wheel profile are captured by the CCD camera together with the optical lens and linear semiconductors laser when the train passes by the device location. The system records the historical data for further analysis. The automatic non-contact-based measured wheelset parts are the inside thickness of the rim and the flange thickness. The system after a certain period of time is supposed to be calibrated to eliminate errors. The system does not still allow the driver of train to be informed about the wear status of the flange thickness. It is also expensive to install it along the long distance of track for more measurements at different sections to detect the track section that is more influential to the wheel wear including the wheel flange thickness reduction. 

Other than track side measurement tools, in early years, the onboard ultrasonic system to detect the wheel flange contact with the rail was developed [31]. The dynamic measurement for the diameter of a train wheel based on structure-light vision was also developed in [32]. The system uses the four structured light sensors, one for the axle and other three for the wheel tread. This technology was more advanced than the use of cycloid constraints with 3-D structured light vision. The measurement method was based on image processing to find out the rolling radius and the contact point between the wheel and the rail. Here, the researchers overlooked the detection through historical records, data validation for future analysis, vibrations on the track and the high cost of the system. 

### 1.3. Conclusions

Among the aforementioned technologies, computer vision-based systems using cameras or lasers and image processing are the only systems which are able to take the wheel flange measurements while the train is in operation. However, these systems are costly in terms of equipment and installation along the whole track line. Therefore, they are not affordable for many railway companies, particularly those in the developing countries. For safety assurance, there is a need to instantly acquire the wheel flange wear, to instantly get informed of the hazardous dimensions of the wheel flange thickness, and to localize the track section that contributes most to the flange thickness reduction for any railway. Therefore, to overcome these points overlooked by the previous researchers, an on-board measurement system is proposed to measure the wheel flange thickness wear in real time, using an inductive displacement sensor. It consists of mechanical parts and electronic parts. The mechanical parts cover the sensor holding fixture. The electronic equipment are grouped into four units: sensing unit, noise removal unit, data acquisition system unit, and display unit. 

## 2. Materials and Methods

The proposed on-board measurement system is based on inductive displacement sensor detection technology. The intelligent measurement system is an automated tool used to measure the physical quantities automatically, analyze data and display the data outputs without any other assistance [33]. Our proposed system takes the measurements of wheel flange thickness wear using shielded active inductive displacement sensor in real-time during railway vehicle motion. The sensor outputs s voltage which is converted into a distance between the sensor position and the flange. The relation that is used to convert the voltage into distance is predetermined using manual measurements with a digital voltmeter and a micrometer. This relation is used in the data acquisition configuration as a scaling and calibration equation. The flange reduction thickness is obtained as a difference between initial and current lateral distances from the flange outer surface and the sensor tip. During experiments, LabVIEW software was used together with the data acquisition (DAQ) interface and a NI USB 6221 board to record the sensor measurements data. With the knowledge of current flange thickness reduction, the current flange thickness is communicated to the railway operators such that they are notified when the hazardous status of the wheel flange thickness is reached. Also, the faulty track section can be identified using the recorded data by the system and the operational control center data, which consist of train positions versus time, and which are obtained by a global positioning system (GPS). The system consists of an inductive displacement sensor, a voltage power supply, equipment that process signals from the sensor, an analog to digital converter, storage devices and displayers. The working principle and optimization is based on the system construction and does not require the start and stop operations [34].

**Figure 1:**
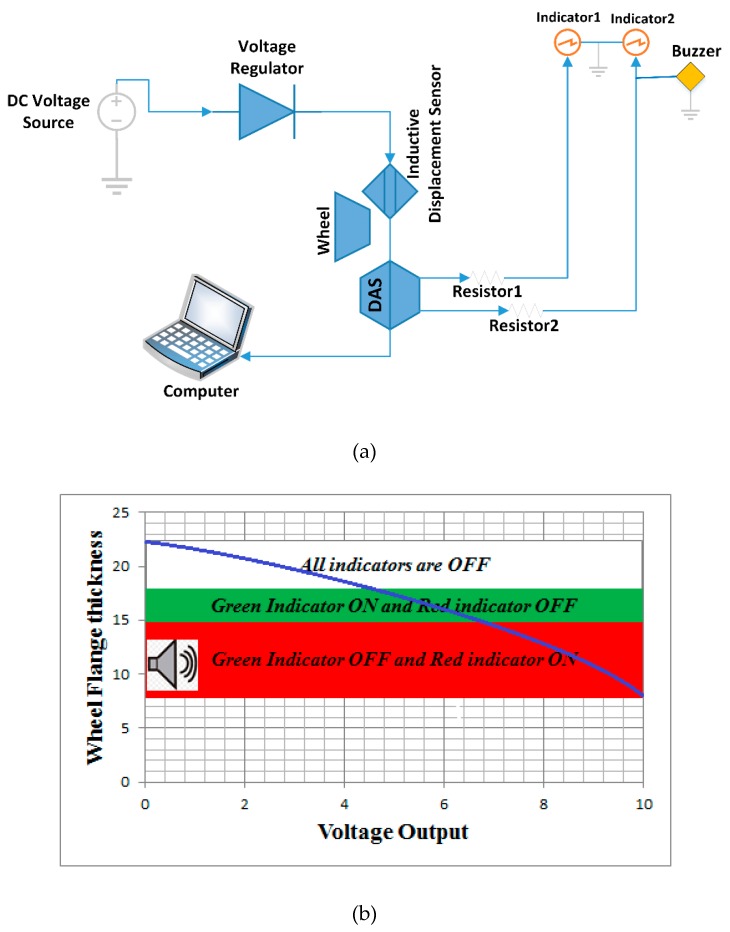
(**a**) Schematic diagram of intelligent system used to measure the wheel flange thickness. (**b**) Wheel flange thickness status in train’s cab.

This on-board intelligent system operates in three different modes:

(1) The wheel flange thickness status data is recorded based using data acquisition system (see Figure 1a). In this mode, the active inductive displacement sensor in proximity to the wheel flange detects the reduction of the wheel flange thickness. The data from the sensor are processed and recorded on a computer. The processing of read data, the settings, and the recordings are done in the LabVIEW software. The data can be displayed in graphical format and/or tabular format. In addition, the data can be analyzed by railway engineers by using their preferred appropriate software.
(2) The measurement output data obtained through the DAQ and LabVIEW codes are visualized in the train cab dashboard by two light emitting diodes based indicators (indicator 1 and indicator 2 as shown in Figure 1) to indicate the status of the wheel flange (see Figure 1). The first indicator notifies the half thickness reduction of a wheel flange (18.5 mm). It uses green color through its light emitting diodes (LEDs). The second indicator displays the minimum dimensions of the wheel flange thickness (15 mm) and the red color from the LEDs is used for this case (e.g., see Figure 1b).
(3) Emergency short time data storage is done in case of the malfunction of the data acquisition system or the computer corruption. The USB cable connects the Arduino board to a computer and transfer data directly to a computer and retains all records. The Arduino code is used to monitor the working operation and the “PLX-DAQ” application software links Arduino code instructions with excel sheet by its macro. Therefore, both mode 2 and mode 3 share common interoperability software and the hardware printed circuit board.

### 2.1. The Inductive Sensor-Based Measurement Method, Mechanics and Design

**Figure 2:**
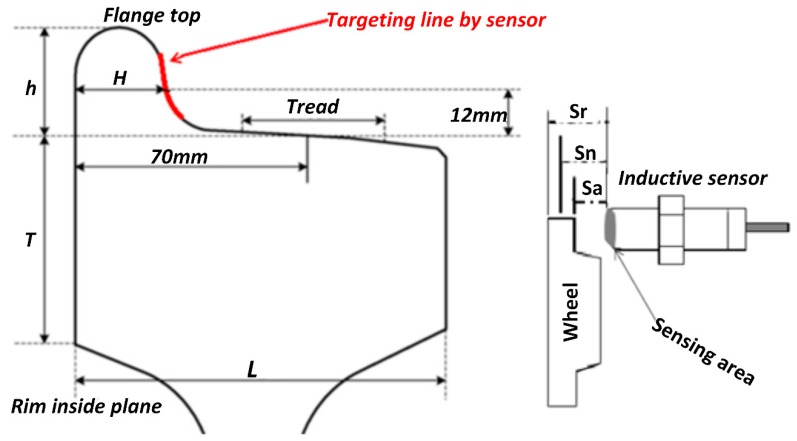
Detected object and sensor characteristics.

The distance between the sensor probe and the wheel flange is the assured sensing distance ($S_{a}$). The train is allowed to operate when the assured sensing distance is less than nominal sensing distance ($S_{n}$). Sensed distance is the displacement between the flange front surface and the sensor probe. The sensor detects the surface wheel flange which is in front of the rail head gauge corner through its sensing area or active zone of the inductive sensor. The sensor probe is in proximity with the flange surface and the position of the flange surface changes in the nominal sensing distance ($S_{n})$. The assured sensing distance ($S_{a}$) or operating range between the wheel flange and sensor probe increases with the thickness of the wheel flange reduction (see Figure 2).

Therefore, the reduced thickness of the wheel flange ($R_{t}$) is the difference between the assured sensing distance and the reference sensing distance ($S_{rf}$). The reference distance of the next day is the assured sensing distance of the previous one. The flange thickness should be located within the nominal sensing distance ($S_{n}$) with its original thickness (H).

The wheel flange is made of steel material which has a good correction factor *(**f**)* of the inductive sensor:

(1) $$S_{n}=f\times d,$$

(2) d=0.25×D

(3) $$B=\frac{\mu_{0}i}{2\pi S_{a}},$$

(4) $$V=\frac{1}{C}\overset{t_{f}}{\underset{t_{0}}{\int }}idt+V_{0},$$

where at $t_{0},\;S_{a}=S_{rf}=V_{0},$ and $\mathrm{at}\;t_{f},\;S_{a}=S_{rf}+R_{t},$ *B* is the strength of magnetic field in Tesla, $\mu_{0}$ is the permeability of the free space, ***i*** is the current in the coil in Ampere, ***d*** is the sensor probe area diameter in mm, $S_{rf}$ is the referencing distance, ***D*** is the detected surface diameter of the sensing object in mm, $R_{t}$ is reduced thickness. 

The high frequency bounce and torsional vibration signals are easily filtered from the sensor output signal as explained in the corresponding “Methods and Materials” and “Results and Discussion” sections.

The output signals are recorded as displacements **(*mm*)** or voltages (***V***) by the data acquisition system (DAS). From Figure 1b, 0.5 Volt corresponds to 18.5 mm from the sensor tip position which is indicated by green light from LED. The maximum voltage is related to the allowed minimum wheel flange thickness (a distance of 15 mm from the wheel flange surface to the sensor tip position). The thickness of the wheel flange which is below a minimum allowable value is indicated by the red light from LED. The flange status can therefore be communicated to the railway operators by means of lights, buzzers, emails, etc. In the case of AALRTS rail vehicles, the allowable wheel flange operation thickness is between 15 mm and 22 mm.

Generally, determining the sensor probe size and the operating range requires the knowledge of the size and the shape of a targeted object [35]. The profile of the rail gauge corner and the wheel flange surface are not flat. Their shapes are designed to reduce the friction between them [26]. Therefore, the surface of the wheel flange front side to be detected by the sensor is convex and its factor on the sensing area is 0.25. The sensor size needs to be as large as possible to increase the practical sensing distance that can work on the convex shape. 

#### 2.1.1. Quantifying the Effect of the Wheelset Dynamics at Curves

**Figure 3:**
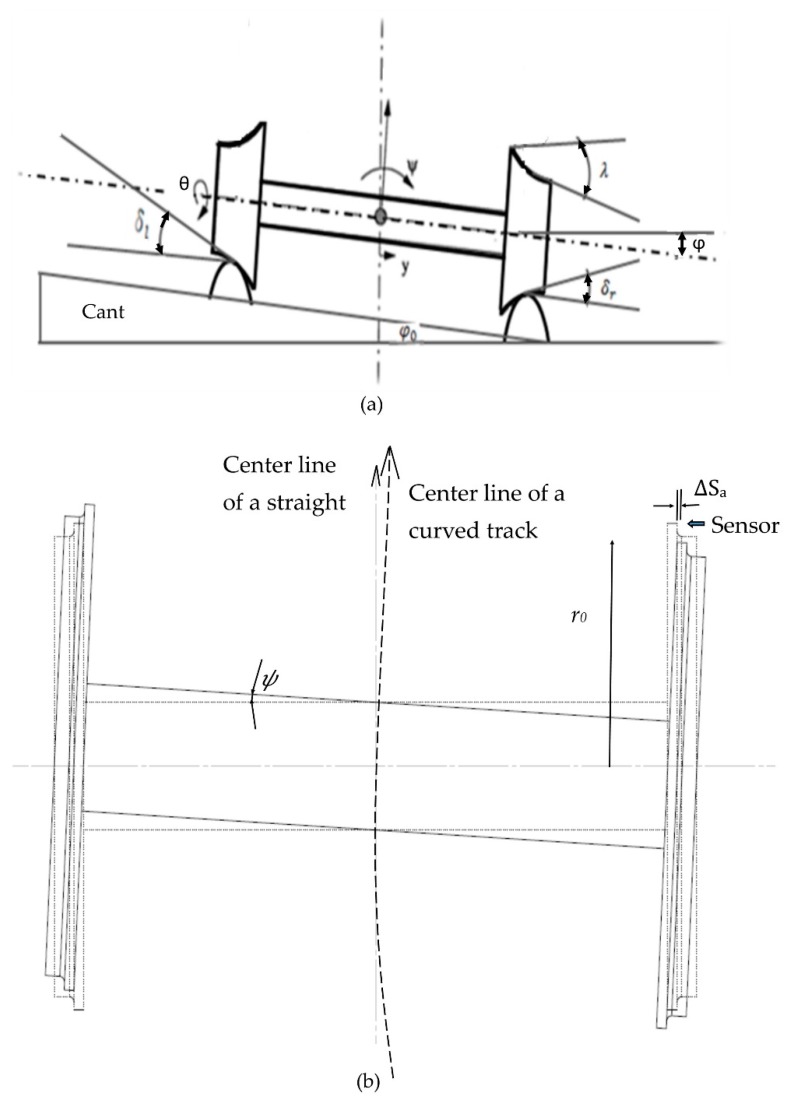
(**a**) Front view of the wheelset at curve with a cant of $\varphi_{0}.$ (**b**) Plan view of the wheelset. Dashed lines represent the wheelset in centered position, and the continuous lines represent the yawed wheelset by an angle ψ.

The wheelset dynamics is expected to introduce low frequency noises in the measurements caused by the wheelset yaw motion at curves. Therefore, the signal contributions from lateral displacements and yaw displacements of the wheelset must be determined. For example as shown in Figure 3b, when the wheel yaws by a small angle ψ, which is the case at curves, the wheel flange deviates from the sensor position by a lateral distance $\Delta s_{a}\approx r_{0}\psi ,$ where $r_{o}$ is the nominal radius of the wheel. With the knowledge of the track geometry specifications such as curve radii, cants, and the rail vehicle specifications, the yaw and lateral displacement of the wheelset can be predicted at the railway line, by solving the governing equations of motions. Such equations have been derived in the reference [36]. The equations are derived by considering the balance of all forces and moments acting on the wheelset (including the creep forces, applied external forces, gravitational forces and corresponding moments) with lateral and yaw inertial forces and torques, and assuming small lateral displacement, such that the wheel rail contact angle on left and right side are approximately equal, $\delta_{l}=\delta_{r}=\delta_{0}$ and $\varphi =\varphi_{0}$ (see Figure 3a to localize these angles).

This assumption is valid if the curve radius is quite large. Assuming the nonlinearity terms are eliminated the equations of motion of the wheelset at curve are given by:

(5) $$m\ddot{y}+\frac{2f_{22}\dot{y}}{V}+K_{y}y-2f_{22}\psi +\left(\frac{2f_{23}}{V}-\frac{I_{y}KV}{r_{o}l}\right)\dot{\psi }=-\frac{mV^{2}}{R_{0}}+mg\varphi_{0}+\frac{\sigma I_{y}V^{2}}{R_{0}r_{0}}+\frac{2f_{23}}{R_{0}},$$

(6) $$\frac{2f_{11}\lambda_{0}ly}{r_{0}}-\left(\frac{2f_{23}}{V}-\frac{I_{y}\delta_{0}V}{r_{0}l}\right)\dot{y}+I_{z}\ddot{\psi }+\frac{2f_{11}l^{2}\dot{\psi }}{V}+K_{\psi }\psi =\frac{I_{y}V\dot{\varphi_{0}}}{r_{0}}-\frac{2f_{11}l^{2}}{R_{0}},$$

where all variables are defined as in Table 1. 

Equations (5) and (6) can be written in a compact form as:

(7) $$\left[M\right]\left\{\ddot{X}\right\}+\left[C\right]\left\{\dot{X}\right\}+\left[K\right]\left\{X\right\}=\left\{F\right\},$$

where $\left\{X\right\}=\left\{\begin{array}{c} y\\ \psi \end{array}\right\},$ $\left\{\dot{X}\right\}$ and $\left\{\ddot{X}\right\}$ are respectively first and second derivatives of {X}:

$$\left[M\right]=\left[\begin{array}{cc} m & 0\\ 0 & I_{z} \end{array}\right],\;\left[C\right]=\left[\begin{array}{cc} \frac{2f_{22}}{V} & \left(\frac{2f_{23}}{V}-\frac{I_{y}KV}{r_{o}l}\right)\\ -\left(\frac{2f_{23}}{V}-\frac{I_{y}\delta_{0}V}{r_{0}l}\right) & \frac{2f_{11}l^{2}}{V} \end{array}\right],\;\left[K\right]=\left[\begin{array}{cc} K_{y} & -2f_{22}\\ \frac{2f_{11}\lambda_{0}l}{r_{0}} & K_{\psi } \end{array}\right],\;\mathrm{and}\;\left[F\right]=\left[\begin{array}{c} -\frac{mV^{2}}{R_{0}}+mg\varphi_{0}+\frac{\sigma I_{y}V^{2}}{R_{0}r_{0}}+\frac{2f_{23}}{R_{0}}\\ \frac{I_{y}V\dot{\varphi_{0}}}{r_{0}}-\frac{2f_{11}l^{2}}{R_{0}} \end{array}\right].$$

Also, Equation (7) can be transformed into a state space representation by performing the following manipulations:

Let choose the state variables $Z=\left\{\begin{array}{c} Z_{1}\\ Z_{2} \end{array}\right\},$ where $Z_{1}=\left\{X\right\}$, and $Z_{2}=\left\{\dot{X}\right\}$. Therefore, ${\dot{Z}}_{1}=Z_{2}$ and ${\dot{Z}}_{2}=\left\{\ddot{X}\right\}=-{\left[M\right]}^{-1}\left[K\right]Z_{1}-{\left[M\right]}^{-1}\left[C\right]Z_{2}+{\left[M\right]}^{-1}\left\{F\right\}.$

The state space standard form of the system equations is written as:

(8) $$\dot{Z}=AZ+BU,$$

y=CZ+DU,

where $A=\left[\begin{array}{cc} 0_{2,2} & I_{2,2}\\ -M^{-1}K & -M^{-1}C \end{array}\right],\;B=\left[\begin{array}{c} 0_{2,2}\\ M^{-1} \end{array}\right],\;U=F,$ **Z** is the state vector, **A** is the amplification gain matrix, **B** is the actuation gain matrix, **U** is the forcing vector or control vector, **Y** is the system output vector, **C** is the measurement gain matrix and **D** is the feedforward gain matrix, $0_{2,2}$ is a 2 by 2 zero matrix and $I_{2,2}$ is a 2 by 2 identity matrix. 

The values of all parameters and constants required to solve the equations are presented in Table 1. From the state space Equation (8), we can solve yaw angle and lateral displacement using analytical or numerical methods. The boundary conditions have been settled as initial displacements and velocities of the variables. The yaw and lateral displacements are solved using the software MatLab. The lateral displacement of the wheelset center does not affect the sensor readings because it follows the center of the wheelset, but the yaw displacement does.

#### 2.1.2. Kinematic Constraints of the Sensor Holding Fixture 

**Figure 4:**
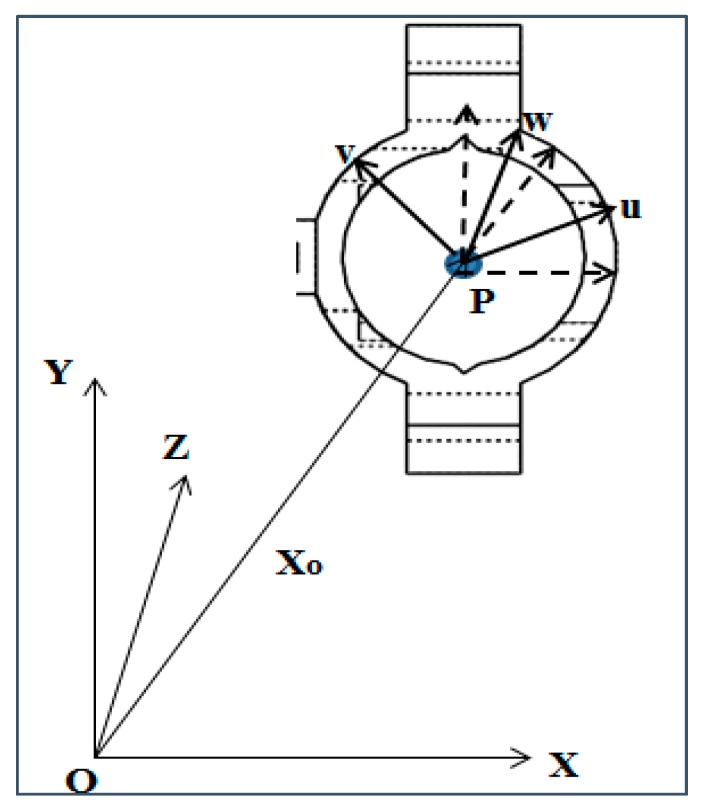
Holding fixture center positioning (**O**: Center of joining the supports and holding clamp (Fixed center) and **P**: Center of the holding clamp circle (moving center))**.**

The sensor holding fixture has two center points that define its operation. One center point is fixed and located at the joint of the two supports and the holding clamp. The second center is a moving center that holds the clamp. All of them should have the positions that keep the inductive displacement sensor in proximity of the wheel flange surface, in front of the railhead gauge corner. As it is shown in Figure 4, each center has three axes to define the sensor working zone and coordinates [37]. 

The first center is the intersection of two holding parts, which are joined by weldment. It is the reference from which the location of the free or moving center in the holding clamp is determined. The fixed center ensures that the moving center is in a position of the sensing zone as constrained by the fixture parts. The position of the moving center in holding clamp can’t allow the sensor to detect other objects out of the assured sensing zone and from its guiding ring.

The holding clamp surface points are defined by a differentiable function, C (u, v, w)=0 or could be represented as C (U)=0, where U is the vector containing all the three axes, u, v and w. All the points that lie outside the surface are defined by C (U)>0. Based on Figure 4, it can be seen that there are two co-ordinate systems: fixed coordinate system of the assembly of the supports with the holding clamp: O (X, Y, Z), and the coordinate which is fixed at the center of the holding clamp: P (u, v, w). The origin of the coordinate system P (u, v, w) is related to O (X, Y, Z) by a distance vector $X_{o}={\left[X,\;Y,\;Z\right]}^{T}$ and an orientation $G={\left[G_{\alpha },\;G_{\beta },\;G_{\gamma }\right]}^{T}$. The coordinate transformation from U to X is given by $X=A\left(G\right)U+X_{0}$. Consider a system of holding clamp elements from 1 through m. The $i^{th}$ element is disturbed by the frequency of vibration:

(9) $$C\left[A{\left(G\right)}^{T}\left\{X_{i}-X_{0}\right\}\right],$$

(10) $$C_{i}\left[q\right]=C\left[A{\left(G\right)}^{T}\left\{X_{i}-X_{0}\right\}\right],$$

where ***q*** is located in O (X, Y, Z). Let $Q={\left[X,\;Y,\;Z,\;G_{\alpha },\;G_{\beta },\;G_{\gamma }\right]}^{T}$. The transformation matrix for 2-D system is given by:

(11) $$\left[\begin{array}{c} u\\ v \end{array}\right]=\left[\begin{array}{cc} cos\left(G_{\alpha }\right) & sin\left(G_{\gamma }\right)\\ -sin\left(G_{\alpha }\right) & cos\left(G_{\gamma }\right) \end{array}\right]\left[\begin{array}{c} X-X_{o}\\ Y-Y_{o} \end{array}\right].$$

When ***Q*** is a unique position where all the clump elements 1 to m are positioned in the sensing zone and facing the targeted line of the flange thickness,

(12) $$C_{i}\;\left[q*\right]=\;0,\;for\;1\leq i\leq m.$$

When the holding clamp is placed in the vicinity ***q***∗ +∆q, the uniqueness of the assured sensing zone together with the guide ring in vicinity of ***q***∗ needs to fulfil the relation:

(13) $$C_{i}\left[q^{*}+∆q\right]=C_{i}\left[q^{*}\right]+T_{i}∆q=0,\;\mathrm{for}\;1\leq i\leq m,$$

where $T_{i}$ is the 1×6 gradient vector consisting of partial derivatives of function $C_{i}$ with respect to *q*,

(14) $$T_{i}=\left[\frac{\partial C_{i}}{\partial X},\;\frac{\partial C_{i}}{\partial Y},\frac{\partial C_{i}}{\partial Z},\frac{\partial C_{i}}{\partial G_{\alpha }},\frac{\partial C_{i}}{\partial G_{\beta }},\frac{\partial C_{i}}{\partial G_{\gamma }}\right].$$

The matrix form of m simultaneous equations is written as:

(15) $$C=\left[\begin{array}{c} \begin{array}{c} T_{1}\\ .\\ . \end{array}\\ T_{m} \end{array}\right],...C∆q=0.$$

For 2-D system, a sample C matrix is:

(16) $$C=\left[\begin{array}{ccc} \frac{\partial C_{1}}{\partial X} & \frac{\partial C_{1}}{\partial Y} & \frac{\partial C_{1}}{\partial G_{\alpha }}\\ . & . & .\\ . & . & .\\ . & . & .\\ \frac{\partial C_{m}}{\partial X} & \frac{\partial C_{m}}{\partial Y} & \frac{\partial C_{m}}{\partial G_{\alpha }} \end{array}\right]$$

For unique holding clamp position, where CΔq = 0, the Jacobian matrix C must have full rank.

#### 2.1.3. CAD Model of the Designed Sensor Holding Fixture 

The sensor holding fixture is built of three main parts which are the horizontal support, the vertical support and the holding clamp. The CAD model of the fixture is designed in consideration of the manufacturing aspects and requirements. 

**Figure 5:**
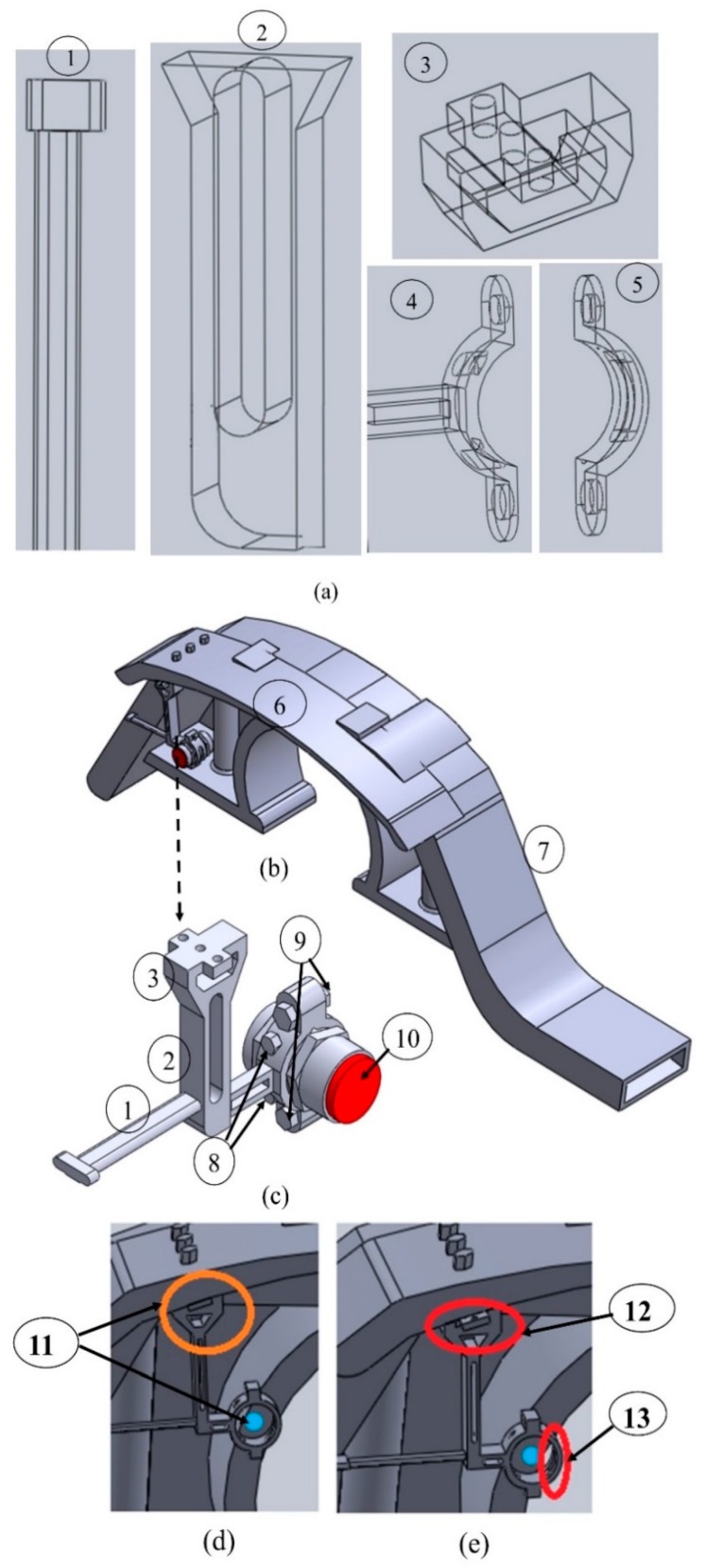
(**a**) CAD model of the parts of the sensor holding fixture, (**b**) CAD model sensor holing fixture assembled on the bogie frame through bogie fender. (**c**) CAD model of sensor holding fixture (complete view). (**d**) Calibrated sensor holding fixture. (**e**) Uncalibrated sensor holding fixture.

As the bogie frame is made in steel, the material properties of the sensor holding fixture must be compatible with the bogie frame to facilitate the joining method used. Hence, all parts are made of steel materials. The vertical support is assembled with the sensor holder and bogie fender by bolt joining method. The horizontal support is joined with the bogie rib by welding method. The holding clamp has two separate parts which are assembled together by bolts to fix the sensor inside them. On the other hand, it is assembled with the supports at fixed center by welding (see Figure 5a). The center of holding clamp changes according to the diameter and the shape of the inductive displacement sensor. 

Therefore, all the fixture parts have specific shapes to make the sensor holding fixture fit in the space between bogie frame rib and bogie fender while keeping the sensor in proximity to the wheel flange front surface. The threaded holes of holding fixture at the top of vertical support have same radii as the radii of the holes at the bogie fender to support the bolts during assembling, as seen in Figure 5b. There are three washers with 3 mm of thickness used at each hole between holding fixture and the nuts at the bottom side. The washer thickness can be varied during fixture calibration.

The nomenclature in Figure 5 presents the names of different parts of the sensor fixture and its surroundings as follows: (1) is the horizontal support; (2) is the vertical support (part 1); (3) is the vertical support (part 2); (4) is the sensor holding clamp (part1); (5) is the sensor holding clamp (part 2);(6) is the bogie fender; (7) is the bogie frame; (8) represents the bolts and nuts for adjusting the sensor in a holding clamp; (9) represents the bolts and nuts for assembling the holding clamp parts and fix the sensor in the holding clamp; (10) is the sensor in the holding clamp; (11) represents the corrected vertical and horizontal adjustment; (12) represents loosen horizontal adjustment; (13) represents loosen vertical adjustment.

The two holes on the holding clamp up and down are not threaded and have the equal diameters to be able to support the bolts used during assembly as seen in Figure 5a. There is one washer at each side of a clamping part to make a good tight and re-tight of the two parts. The two bolts with their nuts to assemble the clamp parts are tightened to a specific torque that cannot damage the sensor housing and the fixture body. According to the sensor diameter and shape the two adjusting bolts are used to keep it in a defined position.

### 2.2. Laboratory Experiments

**Figure 6:**
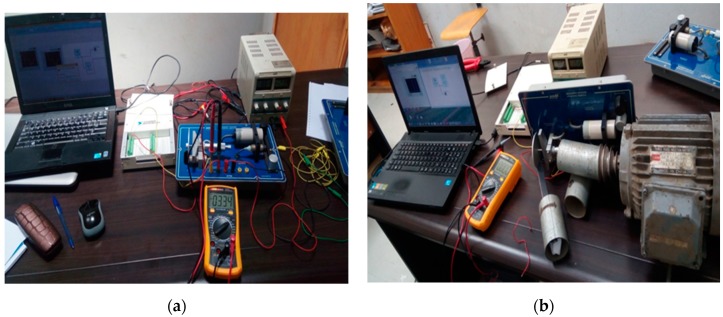
(**a**) Setup for calibration. (**b**) Laboratory setup where complete data acquisition system is mounted to perform various experiments.

We have designed an experimental prototype that mimics the proposed measurement system hardware integrated with the software that is planned to be used in actual measurement system. The purpose of the prototype is to provide evidence that the proposed measurement system will provide accurate measurements. The prototype consists of a rotating disk in contact with a grinder to represent the wheel flange in contact with the rail. An inductive sensor with a capability of measuring 0 to 5 mm distance (4 Volts to 10 Volts) in front of the tip is used. A data acquisition system (DAS) that consists of NI 6221 USB, a computer, and LabVIEW and the DAQ software is interfaced to the inductive sensor. The calibration of the system is done by performing manual measurement of the sensor reading using a micrometer and voltmeter as the disk displaces back and forth in front of the inductive sensor (see Figure 6a). The complete measurement laboratory demonstration setup is shown in Figure 6b. 

## 3. Results and Discussion

This section lays out the results obtained by the methods discussed in the preceding sections. It includes the results of wheelset dynamics, sensor fixture vibration analysis, and laboratory experiments.

### 3.1. Wheelset Dynamics Results

**Figure 7:**
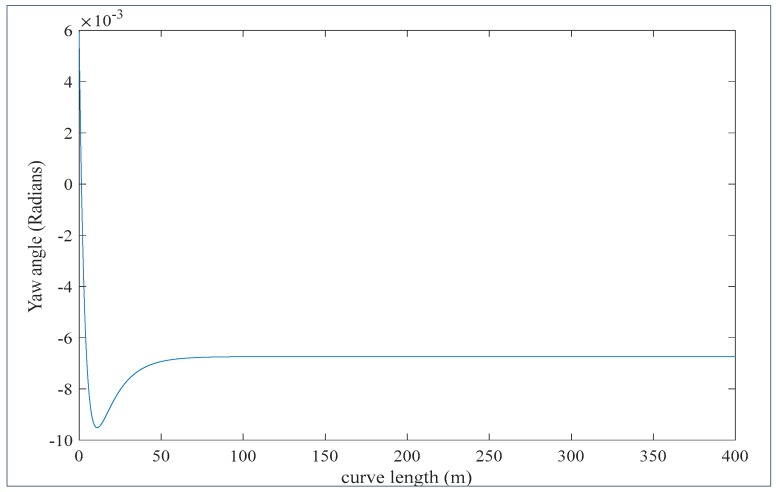
The yaw angle with respect to curve length.

**Figure 8:**
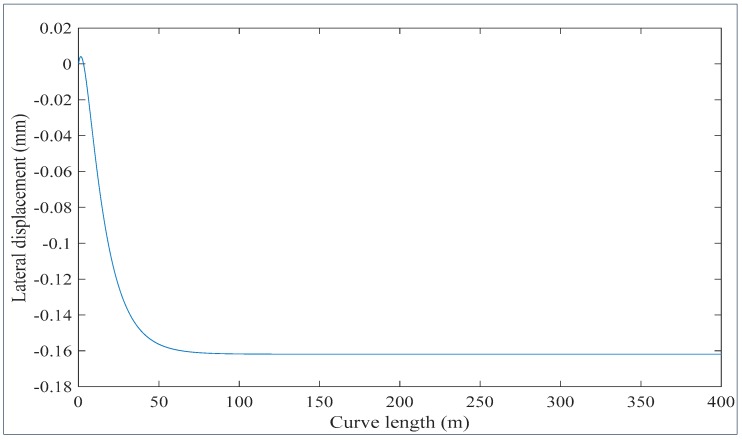
The lateral displacement with respect to curve length.

The state space Equation (8) has been solved using the parameter values listed in Table 1. It is assumed that the rail vehicle travels at a speed of 17 m/s. The results are shown in Figure 7 and Figure 8. Referring to Figure 7, the railway vehicle is yawing at a small angle varying between 0.006 radians and −0.0095 radians or between 0.34 degrees and −0.55 degrees on a curve of 300 m radius. This fact results in a sensor reading error between 2.5 mm and −3.9 mm. This error range is significant as the wheel flange nominal thickness is around 18 mm. Assuming the sensor is attached at the axle side that corresponds to negative yaw motion (see Figure 3), the actual assured sensor reading is obtained by subtracting the error from the sensor reading. Referring to Figure 8, it is seen that when the train approaches the curve the yaw angle become large initially and then afterwards reduces to reach a steady state value at the curve. 

The reason being that at curve there is a cant angle $\phi_{0}$. At the beginning of the curve the train tends to move laterally (see Figure 8) while at the same time the effect of the cant angle, lateral suspensions and torsional suspensions adjust the train motion be centered in the track. However due to the effect of gravitational forces at the curve the train remains with steady state small lateral displacements. The lateral displacement causes the right and left side wheels to run on the rails with different radii, and therefore both wheels rotate at different speeds, which causes a steady state yaw displacement of the axle. The results illustrated in Figure 7 and Figure 8 match well the results obtained by using the Park Stiffly Stable Integration method in [38].

### 3.2. Fixture Vibration Analysis Using Finite Elements

The sensor holding fixture as well as other railway vehicle components attached to the bogie frame are affected by vibrations. It is fixed to the bogie frame by its supports. It has small dimensions and small weight as compared to other components held by the bogie frame. The weight of the holding fixture is equal to  1.7 ×0.395 (kg). The alloyed steel has weight of 0.395 kg per meter [39]. Its mechanism of assembling is mechanically facilitated. Moreover, from different track conditions (rail irregularities, track contaminants, track alignments and geometry) the bogie frame vibrates with up and down oscillations [40]. 

**Figure 9:**
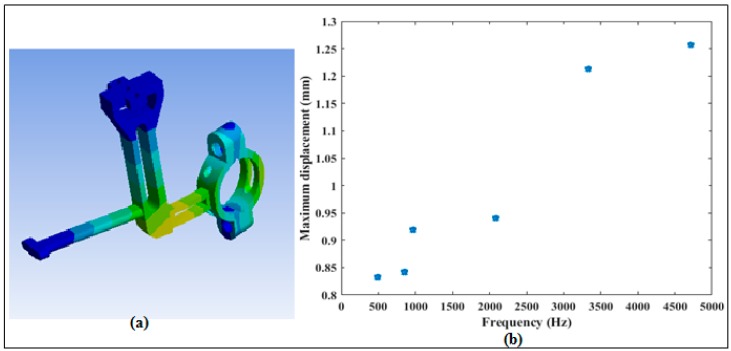
(**a**) FE model of the sensor fixture. (**b**) Frequencies of vibration versus displacements of the sensor holding fixture.

The analyses of vibration has been done using the finite element software ANSYS 15.0. The purpose of the analysis is to observe the frequency content, and their amplitude levels, of the sensor holding fixture caused by vibrations so that they can be considered in the signal filtering method. Random vibrations were applied in the workbench. From Figure 9, we see that the highest frequency of vibration is 4550 Hz at a displacement of 1.27 mm and the lowest maximum displacement is 0.82 mm at 500 Hz of the frequency. These frequency signals are easily filtered as demonstrated by the results in Section 3.3.

For low frequency vibrations, the fixture follows the bogie frame motion and keeps the sensor steady in proximity with the wheel flange but high frequency of vibrations will occur on fixture because it is light and has some elasticity. The displacements in any direction from rigid body motion of the train don’t cause any change in the measurement from sensor. The inductive sensor frequency ranges from 10 to 20 Hz if it is working in alternating current (AC) mode and 500 Hz to 5 kHz working in direct current (DC) mode [41,42].

The vibration frequencies of the sensor holder from the track condition or vehicle conditions can’t exceed the tolerable operating range of the inductive sensor. Therefore, the sensing zone is not affected during the measurement operation.

### 3.3. Laboratory Experimental Results

**Figure 10:**
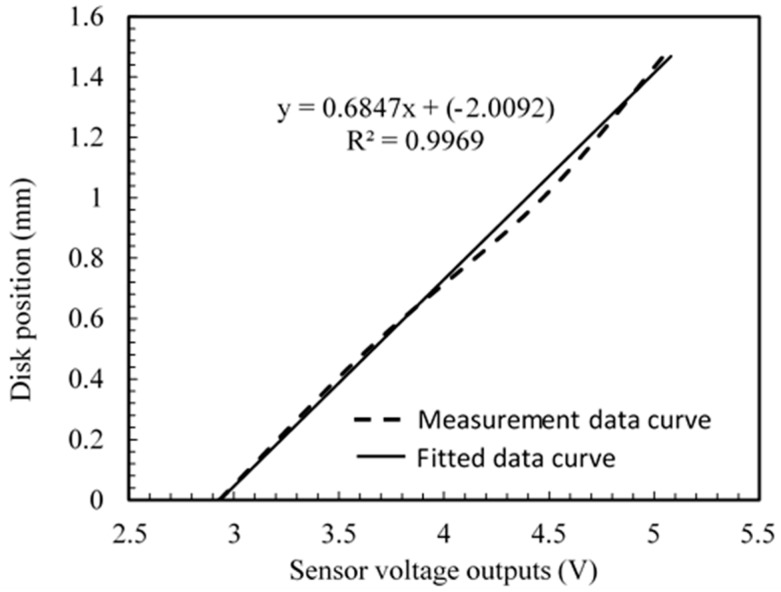
Calibration equation of the inductive displacement sensor from the measurements taken using a voltmeter at the sensor output with respect to the measurements taken using the micrometer as the disk displaces. Input voltage to the sensor is 10 Volts.

**Figure 11:**
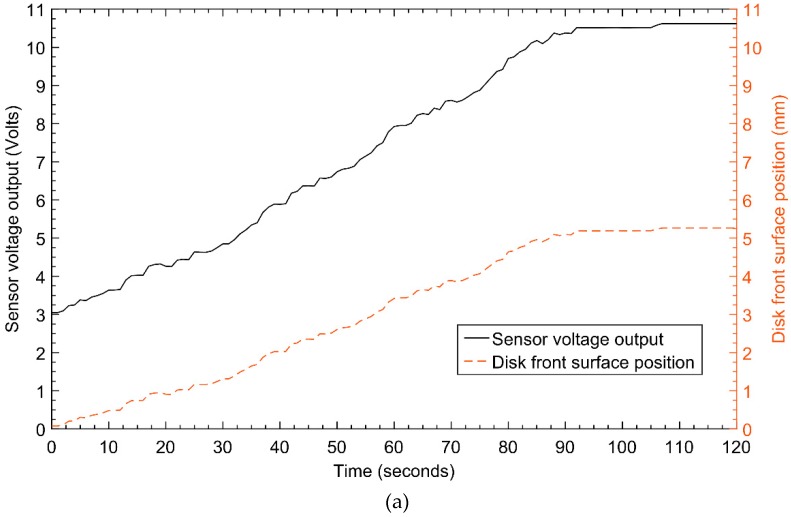

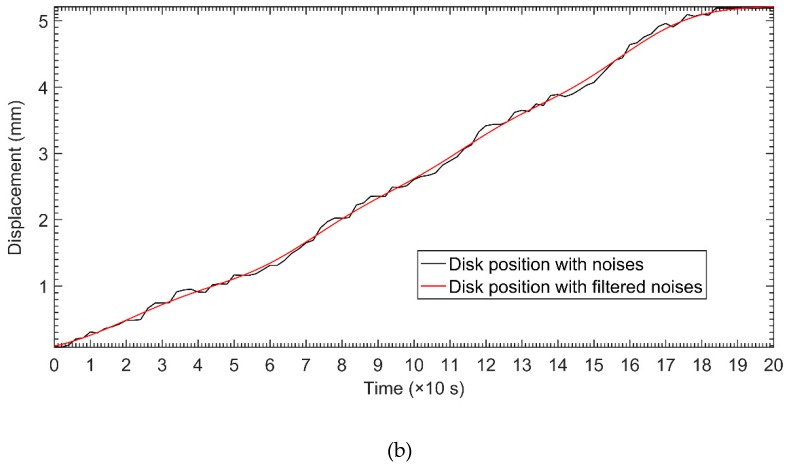
(**a**) Signals of disk front surface position in front of inductive sensor before filtering, in voltage units form and in distance units. (**b**) Disk front surface position measurement with noises and without noises.

The data acquisition system receives the analog signals from the sensor and converts them into a digital signal that processed by the computer software LAVVIEW. The sensor reads the displacement of the disk. The output is displayed in distance units by converting the voltages into mm, using the calibration equation (see Figure 10). In this case, the laboratory simulation is referring to the case of the train motion on the straight line and therefore the recorded signals are not affected by yaw displacements. On the one hand, the hunting motion of the wheelset at straight curve is simulated by the warping of the disk. In Figure 11a, we can observe the two types of signals which are related by calibration equation. The calibration equation is obtained by fitting data that are measured manually by a voltmeter with respect to their corresponding data that are measured by a micrometer, and it is given by:

(17) d=0.6847v−2.0092,

where *d* is the distance in mm, and v is the voltage in volts. This fitting equation achieves a correlation coefficient $R^{2}=0.9969$, which is high enough to assume a perfect linear relationship between the sensor output voltage and distance measured. 

From Equation (17) the sensitivity can derived as the inverse of the slope of the line represented in Figure 10 as:

$$S=\frac{1}{0.6847}\;\mathrm{Volts}/\mathrm{mm}=1.46\;\mathrm{Volts}/\mathrm{mm}$$

The precision of the measurement was also studied by taking repeated measurements four times, all of them at the same conditions and same occasion, but at a different occasion than when the data in Figure 10 and Figure 11 were taken (before six months). These new data are presented in Table 2. The standard deviation was calculated for each repeated value. Then the average of all standard deviations of repeated measurements was calculated to determine the repeatability of the system. The calculated repeatability is ±0.112 Volts, which corresponds to ±0.03 mm by using the sensitivity of the sensor at the time of taking measurements.

This sensitivity is calculated from the data in Table 2 and found to be 3.7 volts/mm. On the other hand the sensor measurement output at different measurement events, spaced by longer period, show that the sensitivity changes significantly. Therefore, calibrations must be done very frequently to maintain its accuracy. 

On the other hand, the resolution of the system can be expressed as the code width of the analogue to digital converter (ADC), which is used in data acquisition system, and can be expressed in terms of code width. NI USB 6221 is proposed and was used in experiment, and its ADC has 16 bits. The code width is given by:

(18) $$Q=\frac{V_{mx}-V_{mn}}{2^{n}},$$

where $V_{mx}$ is the maximum output voltage of data acquisition system, which is 10 Volts; $V_{mn}$ is the minimum output voltage of the data acquisition system, which is −10 Volts; and n is the number of bits used by the ADC, which is 16. Therefore the resolution $Q=20/2^{16}\mathrm{Volts}=0.0003\;\mathrm{Volts},$ and it corresponds to 0.0002 mm for a sensitivity of 1.46 Volts/mm. Thus, a tolerance of 0.0004 mm can be allowed for the measurement system with an assumption that in the worst case the sensitivity will reduce to a half of 1.46 Volts/mm. On the other hand, from the measurements data recorded at AALRTS, the reduction in thickness of the wheel flange from the friction with a rail gauge corner is less than 0.01 mm per day. The total allowed thickness reduction is 7 mm. Therefore the measured thickness reduction plus the reference sensing distance of the sensor must include the nominal sensing distance and allow the minimum tolerance to be ±0.001 mm. This requirement means that the resolution of our instrument should be less than 0.001 mm, which is the case provided as the achieved resolution is 0.0002 mm. 

The results in Figure 11a represent a sample of the measurements taken by the proposed measurement system at the output of the data acquisition system and recorded by the computer. There is a change of assured sensing distance between the sensor probe and disk surface which is recorded as the displacement. The voltage and displacement increase with disk front surface forward displacement away from the sensor. 

At the initial state (t=0), there is a small distance ($d_{0}=0.1\;\mathrm{mm}$) between the probe and the disk surface which corresponds to some voltage called the offset error ($V_{0}=1.95\;V$). The wear removal of disk material would gradually increase and that causes the distance between the sensor probe tip and disk front surface position to increase with time. However, the change in displacements or voltage output does not present a smooth curve.

The displacement curve has some distortions due to the warping of the disk and disk vibrations, which in this case simulates the hunting and vibration motion of the wheelset. Not that the hunting motion of the wheelset induces the yaw motion in the wheelset because of the alterations of the wheelset running radii. Therefore, the low pass filter is used to remove these unwanted signals and allows data acquisition system to keep the correct data for analysis and further study. 

From the results illustrated in Figure 11b, the assured sensing distance increases with respect to the disk thickness reduction from the disk front surface away from the sensor probe tip. The detected physical signal by the active inductive displacement sensor is the position of the disk front surface and reported in terms of displacement. The non-smooth curve in blue color represents the signal recorded by the sensor before passing through the filter. The red line which is very smooth contains the signal after passing through the filter. The frequencies of the smooth signal are between zero and the cut-off frequency of the filter. On the other hand, the material loss of the disk thickness depends on the friction impacted on the disk and other factors such as the torque of a grinder. Therefore by observing the filtered signal we can identify instances where the material loss was significant than other instances. This implies that the recorded signals not only facilitate the monitoring of wheel flange condition but also allows to identify instances that the wear was highly impacted. Therefore comparing the recorded signal of the proposed system DAQ with the train GPS data and timetable data, we are able to identify the section of the track that contribute most to the wheel flange deterioration. 

## 4. Conclusions

In this study a new measurement technology of wheel flange thickness wear is proposed to solve the problem of railway vehicles’ operational safety. It performs measurements in both the static and dynamic state of a train. It is able to record flange thickness wear data versus time. The recorded measurements are useful to monitor in real time the wheel flange thickness range for safe operation. Based on the results obtained during the experiments, the wheel flange wear can be monitored in real-time with a precision of ±0.03 mm. However the system shows that the sensitivity will change over time, but by performing frequent calibration the needed accuracy can be achieved. Therefore, the measurement of wheel flange using the proposed on-board intelligent system can increase railway vehicle operation safety in many curved tracks with small radii by early detection of hazardous wheel flange wear. On the other hand, the proposed system can be used to identify the track sections that can cause a high level of wheel flange wear, to validate and calibrate or update the wheel flange wear evolution predictive models, and to perform analysis as needed by railway operations. The system is commendable to the railway industries for their safety measures and maintenance achievements. It has higher accuracy and precision at low cost as compared to existing multi-camera systems along the railway tracks as well as the on-board laser based systems. 

## Figures and Tables

**Table 1:** The constants used in the wheelset dynamic equations.

S/N	Symbol	Value	Meaning
1	G	1.435 m	The track gauge
2	a or l	0.7175 m	The half of track gauge or half of the distance between contact pints on two rails
3	R	300 m	The radius of curvature
4	m	1250 kg	Mass of the wheelset axle
5	g	$9.81\;N/m^{2}$	Acceleration due to gravity
6	I_z_	$688\;{\mathrm{Kg}\;m}^{2}$	Roll moment of inertia of the wheelset
7	$I_{w}$	$688\;{\mathrm{kg}\;m}^{2}$	Yaw moment of inertia of the wheelset
8	$I_{y}$	$100\;{\mathrm{kg}\;m}^{2}$	Spin moment of inertia of the wheelset
9	$r_{0}$	0.42 m	Nominal radius of the wheel
10	V	17 m/s	Axle speed
11	$f_{11}$	$7.44\times 10^{6}\;N$	Lateral creep force coefficient
12	$f_{12}$	$3120\;{\mathrm{Nm}}^{2}$	Lateral/spin creep force coefficient
13	$f_{22}$	$6.79\times 10^{6}\;N$	Spin creep force coefficient
14	$f_{23}$	$13.7\times \;10^{3}\;\mathrm{Nm}$	spin creep force coefficient
15	$f_{33}$	$2.563\times \;10^{6}$ N	Longitudinal creep force coefficient
16	$k_{y}$	$23\;\times \;10^{4}\;\mathrm{kg}/m$	Lateral stiffness
17	$c_{y}$	0 kg sec/m	Lateral damping
18	k_ψ_	$25\times \;10^{5}\;\mathrm{kgm}/\mathrm{rad}$	Yaw stiffness
19	c_ψ_	0 gm sec/rad	Yaw damping
20	$\sigma =\delta_{0}$	0.0493 rad	Initial tapper angle
21	λ	0.05 rad	Wheel conicity angle

**Table 2:** Repeated measurements of disk displacement by inductive sensor and a micrometer taken at a same occasion. Input voltage to the sensor is 15 Volts.

Micrometer Measurements (mm)	Sensor output Voltage (Volts)			
Same for All Records	1st Records	2nd Records	3rd Records	4th Records
0.5	2.08	2.08	2.04	2.08
1	3.26	3.32	3.23	3.22
1.5	5.28	5.3	5.29	5.29
2	7.29	7.37	7.24	7.2
2.5	9.21	9.18	9.26	9.2
3	11.75	10.75	10.79	10.75
